# Species-Specific Identification from Incomplete Sampling: Applying DNA Barcodes to Monitoring Invasive *Solanum* Plants

**DOI:** 10.1371/journal.pone.0055927

**Published:** 2013-02-07

**Authors:** Wei Zhang, Xiaohong Fan, Shuifang Zhu, Hong Zhao, Lianzhong Fu

**Affiliations:** 1 Institute of Plant Quarantine, Chinese Academy of Inspection and Quarantine, Beijing, China; 2 Marine College, Shandong University at Weihai, Weihai, China; 3 Institute of Botany, Chinese Academy of Sciences, Beijing, China; Biodiversity Insitute of Ontario - University of Guelph, Canada

## Abstract

Comprehensive sampling is crucial to DNA barcoding, but it is rarely performed because materials are usually unavailable. In practice, only a few rather than all species of a genus are required to be identified. Thus identification of a given species using a limited sample is of great importance in current application of DNA barcodes. Here, we selected 70 individuals representing 48 species from each major lineage of *Solanum*, one of the most species-rich genera of seed plants, to explore whether DNA barcodes can provide reliable specific-species discrimination in the context of incomplete sampling. Chloroplast genes *ndhF* and *trnS*-*trnG* and the nuclear gene *waxy*, the commonly used markers in *Solanum* phylogeny, were selected as the supplementary barcodes. The tree-building and modified barcode gap methods were employed to assess species resolution. The results showed that four *Solanum* species of quarantine concern could be successfully identified through the two-step barcoding sampling strategy. In addition, discrepancies between nuclear and cpDNA barcodes in some samples demonstrated the ability to discriminate hybrid species, and highlights the necessity of using barcode regions with different modes of inheritance. We conclude that efficient phylogenetic markers are good candidates as the supplementary barcodes in a given taxonomic group. Critically, we hypothesized that a specific-species could be identified from a phylogenetic framework using incomplete sampling–through this, DNA barcoding will greatly benefit the current fields of its application.

## Introduction

DNA barcoding is a species diagnostic technique using standardized DNA regions across all possible forms of life [1], [2]. This method is promising for taxonomy-related studies owing to its rapid and accurate use with micro materials, for which traditional identification is not feasible. DNA barcoding, using the mitochondrial *coxI* gene (COI), is now well established in animals (e.g.[3]–[5]). However, there is no such single locus to barcode land plants due to the low mutation rate of plant plastid genomes [6]–[8]. In addition, complex evolutionary histories, such as hybridization and polyploidy, are common in plants, and make species boundaries difficult to define [9]–[12]. Thus, multiple genetic loci might be necessarily included in plant barcodes to provide adequate information [13]–[15]. The selection of plant barcode loci involved complex trade-offs between universality and discrimination. The ideal barcodes would require a certain level of variation for discriminatory power. However, they should also be somewhat conservative for universality and ease of alignment. This double standard is a great challenge for the choice and use of a perfect barcode. As a result, a tiered method has been proposed: a first tier composed of a conservative (coding) region shared by all land plants provides resolution at a higher rank (e.g. family or genus) and an additional more variable (coding or noncoding) region provides resolution at species level [16]. Recently, a two-locus combination of *matK*+*rbcL* from the chloroplast genome and the nuclear *ITS* region was successively recommended as the core barcode for land plants [17]–[19]. However, the supplementary barcodes, the choice of which depends on the group itself, are still inconclusive. Therefore, screening and testing supplementary barcodes in certain groups may be an important goal for future DNA barcoding.

One applied field that urgently needs the barcode technique is biomonitoring, in which foreign species are required to be accurately and rapidly distinguished from their close domestic relatives [20], [21]. This process is difficult because the materials used for identification usually lack adequate information and/or sometimes only a fraction of organism is available, thus DNA barcoding will greatly benefit this work. However, to date, applying DNA barcodes to plant biosecurity is challenging. In addition to the problem of the loci chosen in plant barcoding as mentioned above, taxon sampling is another difficulty hindering its rapid use. Quarantine weeds are often exotic species within a large genus distributed worldwide. As a result, constructing a barcode library that includes quarantine weeds and all their relatives, with multiple individuals per species, is extremely difficult. In view of these problems and challenges, one important issue is how to carry out biomonitoring through DNA barcoding without comprehensive sampling.

To date, a large number of taxa have been phylogenetically studied, using efficient markers in sophisticated testing. Can these markers be used as the supplementary barcode regions for a given taxonomic group? If this is possible, it will greatly minimize the amount of work for the next supplementary barcode screening study. More importantly, adding the sequence information of species of interest, such as plants of economic importance, to the existing phylogenetic data matrixes would greatly reduce the sampling work. To some extent this would overcome the sampling difficulty that has always troubled the barcode researcher, especially when studying a large group with worldwide distribution or endangered species with rare materials. This issue, although very important to DNA barcoding, has not been critically investigated.

*Solanum*, with ca. 1400 species distributed worldwide, is the largest genus in the family Solanaceae and within the top ten of the most species-rich genera in seed plants [22], [23]. This taxonomic group contains not only many members of economic importance, such as eggplant and tomato, but also a large number of noxious weeds, among which four species *S. carolinense*, *S. elaeagnifolium*, *S. rostratum*, and *S. torvum* are of great concern as quarantine pests in China and other countries. These four invasive species are a serious threat to the ecological environment and livestock production owing to their strong adaptability and poisonous substances contained. However, they are difficult to remove artificially as the plants are covered with sharp prickles. For these reasons, they have been listed as the most dangerous weeds and are rigorously monitored by quarantine authorities. These species are, however, not easily distinguished from their relatives because of the shortage of reliable characters. This is especially so for quarantine and inspection staff, who mainly work with their seeds.

In this paper, DNA barcoding of *S. carolinense*, *S. elaeagnifolium*, *S. rostratum*, and *S. torvum* was studied in the context of biosecurity. Here, we selected two chloroplast gene regions (*ndhF*, and *trnS*-*trnG*) and one nuclear gene region (*waxy*), which have been widely used in *Solanum* phylogeny, as the supplementary barcode regions. Therefore, most sequences of the DNA regions used in this study are available in GenBank, and only a few species of interest were needed to add to the study. The general aims of the study were to (1) test the feasibility of using efficient phylogenetic markers as the supplementary barcode and (2) develop and test the hypothesis that barcoding of a single (or a few) species of interest could be realized through incomplete sampling. Thus, our results may provide new insights in the current fields of application of DNA barcoding on how to efficiently use existing phylogenetic information to identify specific species.

## Materials and Methods

### Sampling Strategy

Previous molecular phylogenetic studies showed that *Solanum* species can be divided into 13 groups [22], [23]. However, the species of interest are included exclusively in the Leptostemonum group, whose members include most of the spiny *Solanum* species. [24]. According to these results, we used 70 individuals of 48 *Solanum* species, including 31 samples from the present study and 39 from Genbank. These samples represent nine of the 13 groups of *Solanum*, with special emphasis on the Leptostemonum group–of which 35 species were sampled, covering all ten clades of the group [24](Table S1). Most sequences obtained from GenBank were extracted from published articles [22]–[27], and we added a few new individuals of species of interest, especially of the four quarantine species (*S. carolinense*, *S. elaeagnifolium*, *S. rostratum*, and *S. torvum*) and their possible closest relatives documented in existing phylogenetic studies. The materials used were mainly seeds from seed companies outside of China, weeds intercepted by CIQ (China Inspection and Quarantine) authorities, escaping species around import enterprises, and herbaria species exchanged from abroad. Thus, although most of the original sources of materials were uncertain, their diverse obtained sources guaranteed genetic divergence among the species. A potential outgroup of *Jaltomata procumbens* was determined from a previous phylogenetic study [23].

### DNA Extraction, Amplification and Sequencing

Total genomic DNA was extracted using a modified CTAB protocol [28] or plant DNA Extraction Kit (Tiangen Biotech, Beijing, China). The PCR primer and its reaction conditions for *ndhF* region were followed from Olmstead and Sweere [29] and Bohs and Olmstead [25]; those for *trnS*-*trnG* were according to Hamilton [30] and Levin et al. [26]. The *Waxy* region was originally amplified and sequenced using primers 181F and 1171R [31], and *Solanum* specific primers were designed based these sequence (WAXYS: 5′-ACT GCT ATA AAC GTG GGG TTG ATC G-3′; WAXYA2∶5′-TGG AAC CAA CAT AAA ATC AGC-3′). The PCR programs were 94°C for 4 min, followed by 36 cycles of 94°C for 30 s, 53°C for 30 s, and 72°C for 1.5 min, with a single cycle of 72°C for 10 min. PCR products were purified using a Tiangen (Beijing, China) PCR purification kit, and then sequenced bi-directionally on a 3730XL DNA analyzer (Applied Biosystems, Foster City, CA, USA). For some of the *waxy* regions with poor sequence quality, PCR products were cloned with the pGEM-T EASY Vector System II (Promega), with 6–8 clones per individual selected and bi-directionally sequenced with the primers T7 and SP6.

### Data Analysis

Sequence alignments were initially performed with ClustalX [32], and adjusted manually using BioEdit version 7.0.5 [33]. Sequence variation and Kimura 2-parameter (K2P) distance matrix were computed with MEGA 4.0 [34]. Barcoding gaps were evaluated by comparing the inter- and intra-specific genetic divergences [35]. To further show the genetic divergence of each individual, an alternative method was proposed and tested: we compared their K2P distances with each other, analyzed the matrix using a principal components analysis (PCA) module in MVSP (Multi-Variate Statistical Package, http://www.kovcomp.co.uk/mvsp/index.html) and constructed a scatter plot to show the result [36]. Species discrimination was evaluated through tree-based analysis. The Neighbor Joining (NJ) tree recommended as the standard barcoding method [1] was adopted and performed with MEGA 4.0 based on the K2P model [34], and branch support was evaluated with 500 bootstrap replicates. Phylogenetic analyses based on maximum parsimony (MP) were performed using the program PAUP* version 4.0b10 [37]. Bootstrap analyses based on 500 replicates with ten random additions per replicate were used to estimate the confidence of the clades. Unambiguous indels were treated as phylogenetic characters according to the simple indel coding method [38] and performed by GapCoder [39].

## Results

### Character Analysis of Barcode Sequences

Of the 31 individuals used in this study, PCR amplification was successful for all three loci. The PCR production of the *waxy* locus yielded a single band, in which 6–8 clone sequences were identical in the cloned samples, confirming the single copy of *waxy* in *Solanum* as previously reported [40]. We obtained additional *trnS*-*trnG*, *ndhF* and *waxy* sequences of 40 species from GenBank– a total of 213 sequences were used in the present study. The lengths of the aligned DNA fragments of *trnS-trnG*, *ndhF* and *waxy* were 681, 1745 and 1527 bp, respectively. Among the three regions, *waxy* provided the greatest number of variable sites (642) and the highest percentage of both variable characters (42.04%) and parsimony informative characters (21.68%). In addition, this region also showed the greatest mean inter–specific distance (0.0453) for the DNA barcode (Table 1). To get higher discriminatory power, we combined all DNA regions together. The combined matrix ranged from 3760 bp (*S*. *thelopodium*) to 3845 bp (*S*. *macrocarpon*7) in length, and the aligned length was 3953 bp, with 1080 (27.32%) variable characters and 552 (13.96%) informative characters (Table 1). To infer the putative hybrids among the examined species, the cpDNA and nuclear DNA were separately phylogenetically analyzed–when gaps were coded this produced a total of 2480 and 1621 bp alignment, respectively.

**Table 1:** Sequence characteristics of the three DNA regions and their combinations in the studied *Solanum* species.

Statistic	ndhF	trnS-trnG	Waxy	ndhF+trnSG	ndhF+trnSG+Waxy
Length range (bp)	1741–1744	556–610	1450–1503	2297–2353	3760–3845
Aligned length (bp)	1745	681	1527	2426	3953
No. of variable characters (%)	251 (14.38%)	187 (27.46%)	642 (42.04%)	438 (18.05%)	1080 (27.32%)
No. of parsimony informative characters (%)	127 (7.28%)	94(13.80%)	331 (21.68%)	221 (9.11%)	552 (13.96%)
Sequence divergence (Pi)	0.0138	0.0284	0.0453	0.0173	0.0277

### Monophyletic Test Based on Phylogenetic Trees

**Figure 1:**
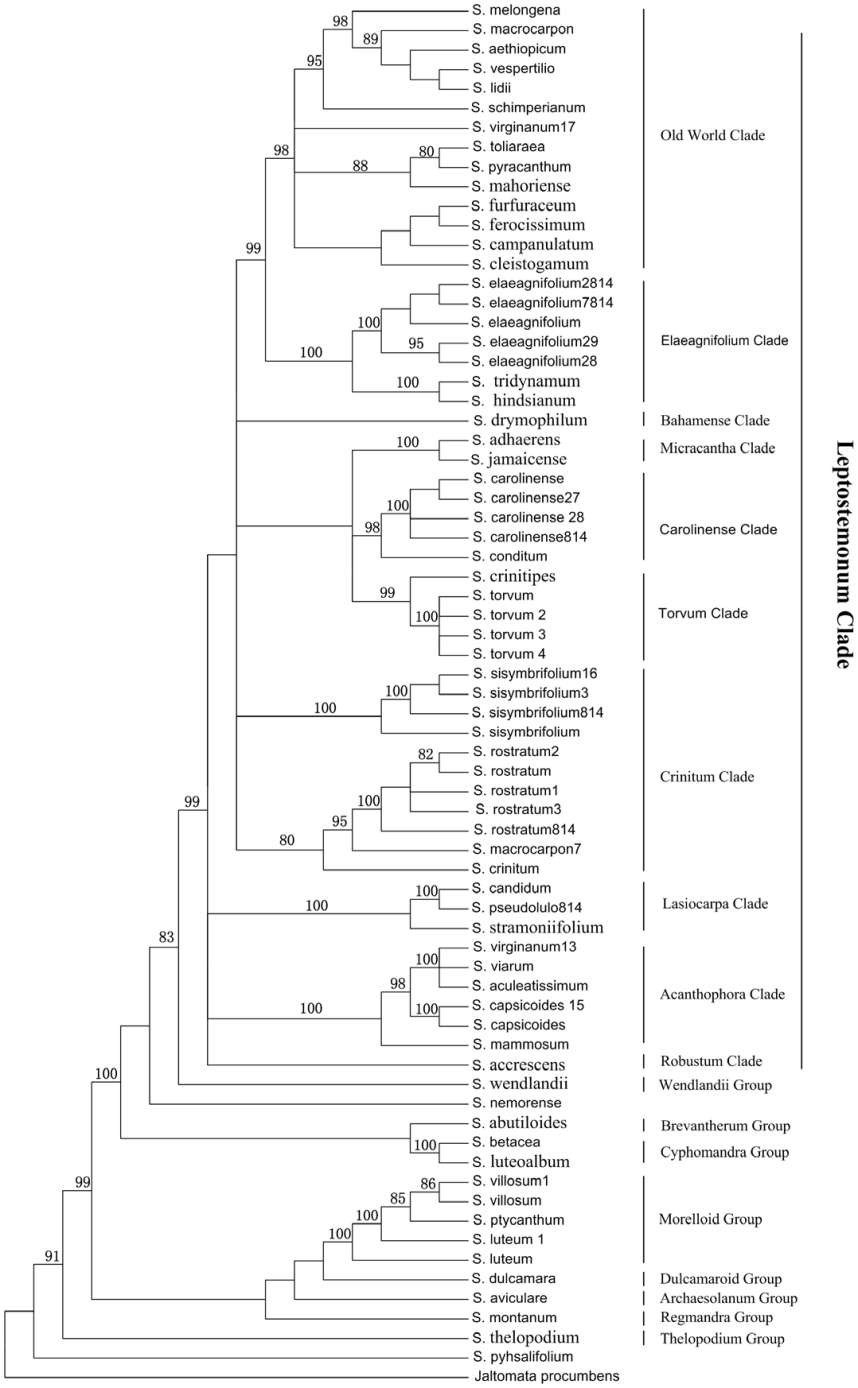
Neighbor joining tree based on the three combined DNA regions (*ndhF*, *trnS*-*trnG* and *waxy*). Bootstrap values (>80%) are shown above the branches. Numbers followed taxon names are individual numbers (see Table S1).

The 71 individuals were divided into nine clades in the NJ tree, and each clade corresponding to a taxonomic group recognized by previous authors (Figure 1). In the Leptostemonum clade, species were subdivided into ten strongly supported clades, concordant with the complete ten taxonomic clades recognized by Levin et al. [24]. The four quarantine species *S. carolinense*, *S. elaeagnifolium*, *S. rostratum and S. torvum* were nested within the carolinense, elaeagnifolium, Crinitum and torvum clades, respectively. In these clades, the four target quarantine species were successfully identified because their individuals were clustered together into a 100% supported monophyletic group, which separated them from their closest relatives. All *Solanum* species with multiple individuals were recovered as monophyletic except those of *S. luteum*, *S. macrocarpon* and *S. virginanum*–especially the later two, which fell into two topologically disjunct but individually well supported clades (Figure 1). In order to explain the reasons for these results, the MP analyses were performed based on the cpDNA and nuclear DNA datasets respectively. In the MP tree of nuclear DNA (*waxy*), the individuals of *S. macrocarpon* divided into two distinct clades, one (*S. macrocarpon*) nested within the old world clade, while the other (*S. macrocarpon7*) fell into the Crinitum clade. However, these two individuals were clustered together in the cpDNA tree, in the same phylogenetic place as that of individual *S. macrocarpon* in the nuclear tree. These incongruent results indicated that the individual *S. macrocarpon7* may be a hybrid species of *S. macrocarpon* (♀) × *S. rostratum* (♂). In contrast, the two individuals of *S. virginanum* were also phylogenetically conflicting but each was consistent in the place of cpDNA and nuclear DNA trees of their own (Figure S1). These results show that at least one of the two individuals was mistakenly identified. In sum, our results showed that the supplementary barcode of *trnS*-*trnG*+*ndhF* (cpDNA)+*waxy* (nuclear DNA) regions in *Solanum* had sufficient discriminatory power to not only identify a given species but also their hybrids.

### Barcode Gap Test

**Figure 2:**
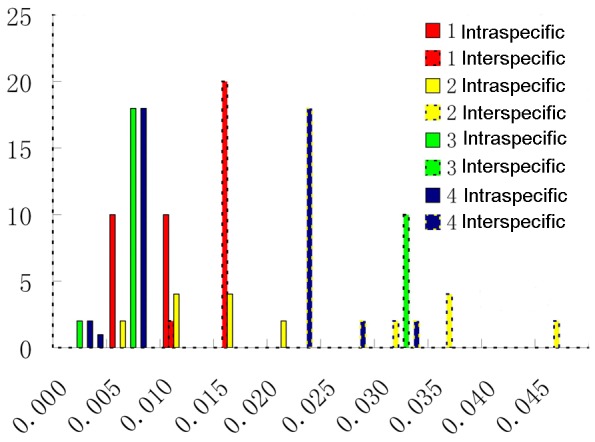
Barcoding gaps between the four quarantine species and their closest relatives. The X-axis relates to the K2P distances of the three combined DNA regions (*ndhF*, *trnS*-*trnG* and *waxy*) between the four quarantine species and their closest relatives. 1. *S. elaeagnifolium*; 2. *S. carolinense*; 3. *S. torvum*; 4. *S. rostratum*. The Y-axis corresponds to the number of occurrences.

**Figure 3:**
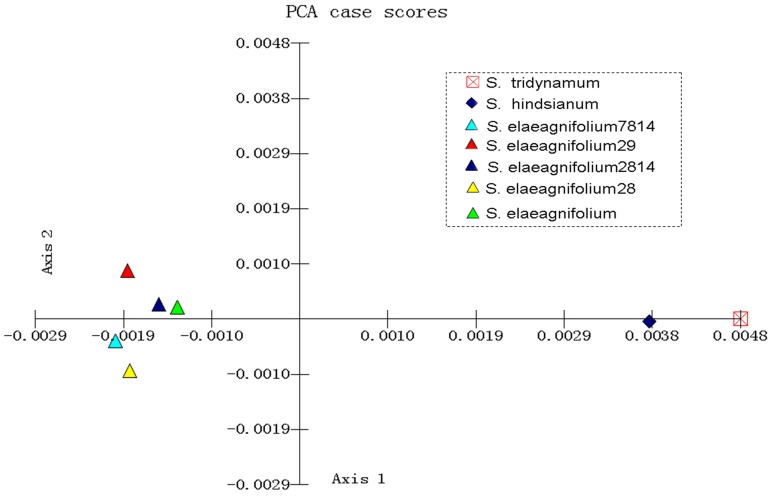
Scatter plot of K2P genetic distances of *S. elaeagnifolium* and its closest relatives.

The barcoding gap enables the assignment of unknown individuals to their species with a negligible error rate. The utility of barcoding is based on the hypothesis that genetic variation between species is much larger than those within species, thus generating a barcoding gap. To date, the barcoding gap has been widely used in well-sampled groups to evaluate all-species discriminatory power of a barcode region. However, few studies have considered the application to specific-species resolution given insufficient sample data. In the present study, we identified four given species using a barcoding gap based on the assumption that a specific species can be identified if it was separated from its closest relative, even though the sampling was insufficient. By comparing the K2P distances, we tested whether the barcoding gap of the three combined DNA regions existed among the four quarantine species and their closest relatives. The results showed that three of the quarantine species had barcoding gaps, the exception being *S. elaeagnifolium*, with an intra- and inter-specific genetic variation overlap (Figure 2). We examined the source data of *S. elaeagnifolium* and its two closest relatives (*S. hindsianum* and *S. tridynamum*) and found that inter-specific genetic variation between *S. hindsianum* and *S. tridynamum* was close to the intra-specific genetic variation within *S. elaeagnifolium*, and thus led to the overlap (Table S2). Moreover, the PCA scatter plot, based on K2P genetic distances, showed that individuals within *S. elaeagnifolium* were more closely related to one another than any were to *S. hindsianum* or *S. tridynamum* (Figure 3). This result demonstrated a genetic gap between *S. elaeagnifolium* and its closest relatives.

*S. hindsianum* and *S. tridynamum.* Thus, the results indicated that the heterogeneous nature of intra- and inter-specific genetic variations across all taxa blurred the barcode gap boundary and thus made all species identified through barcode gaps more difficult (Figure S2). In contrast, the barcoding gap between a given species and its closest relatives was always clear cut and could be used unambiguously to identify specific species (Figure 2 and 3).

## Discussion

Unlike *coxI*, which is well established in animal DNA barcoding, there is no such single region with adequate efficiency to barcode all plant species. Although the two-loci combination of *rbcL*+*matK* and *ITS* have been proposed as the core barcode for land plants, no one marker or their combinations have discriminatory power of >80% [17]. This low resolving power of the core-barcode markers or their combinations limits their use to identifying ‘species group’ (e.g. family and genus) rather than species. As a result, using these markers to identify geographically diverse taxa in a given site where many samples are not necessarily closely related, and/or a large-scale taxonomic group with distantly related species, is workable [41]–[44], whereas, DNA barcoding of closely related species and/or taxonomically complex groups frequently fail [45]. Therefore, it is necessary to develop supplementary barcodes for particularly narrowly circumscribed taxonomic groups. In the present study, we selected the *ndhF*, *trnS*-*trnG* and *waxy* regions – the phylogenetic markers commonly used in *Solanum* studies – as the supplementary barcode to test discriminatory power. Theoretically, powerful phylogenetic markers do not always equate to an efficient DNA barcode, because variable characters of parsimony informative utility are not always the unique changes used as ‘species markers’ [13]. However, in our three DNA regions, the percentage of the variable characters increased with the parsimony informative characters increasing. Accordingly, the sequence divergence also increased with the variable characters. In the NJ tree, all individuals of a single species (except those of *S. luteum*, *S. macrocarpon* and *S. virginanum*, discussed below) clustered together in a monophyletic group. Furthermore, all of the three regions were 100% successful in PCR amplification. These results suggest that the phylogenetic markers are so efficient that they would be good candidates for DNA barcodes.

To date, plant barcodes have been based exclusively on the chloroplast genome. However, hybridization and polyploidy are common in plants [11], [46]–[48], which seriously affects the use of any uniparental inheritance locus for species boundary delimitation. Thus, a combination of DNA markers with different modes of inheritance is necessary in plant barcodes. Although the *ITS* region has been exclusively proposed as a nuclear marker, knotty problems, such as incomplete concerted evolution, fungal contamination and low recovery in some groups reduced its utility [49], [50]. Therefore, it is necessary to add additional nuclear regions as complementary markers in barcoding of a given taxonomic group. In the present study, we used both the chloroplast genes *trnS*-*trnG* and *ndhF* and the nuclear gene *waxy* to explore the species boundary, and the contradiction between different gene trees implied that the individual *S. macrocarpon7* was a hybrid and one sample of *S. virginanum* had been misidentified. These results have universal significance, because both of the phenomena are common in plant systematics [13]. Thus, our results confirmed the importance of using multiple markers from different genomes for plant barcodes.

Taxa sampling, directly related to both the inter- and intra-specific divergence, is critically important in DNA barcoding. Although there is agreement that barcoding performs poorly in incomplete samples [35], [51] how many specimens are needed to construct a reliable reference for species identification is still inconclusive. Some authors suggested sampling 5–10 individuals per species (http://www.boldsystems.org/index.php/Login/page), but this is rarely done [52]. Identification of all species of a taxonomic group using complete sampling has been intensively investigated, but is it necessary to barcode a single or a few species using all congeneric species? If not, what is the minimum number and which is necessary needed? This is rarely assessed. In the present study, we explored a two-step sampling strategy to identify four quarantine species from a species-rich genus comprising ca. 1400 species. The first step was clade-sampling– sampling representive species from each primary evolutionary clade of the genus according to the previous phylogeny. Theoretically, genetic distances within clades are much smaller than those between clades, thus generating a genetic gap between clades. As a result, each clade of the group can be identified. We call this step ‘clade barcoding’. If an unknown species is nested within a given clade, we can then conduct the second step– adding species and individuals in this clade–until the unknown species is nested exclusively within a species’ monophyletic group. Thus, specific species can be identified. In using this sampling method, we densely sampled individuals of the target species and closest relatives, but only selectively sampled representatives of distinct relatives. As a result, a large number of unrelated species were removed from the analysis. This taxa sampling strategy, in combination with the utility of the previous phylogenetic markers, from which many sequence are available in Genbank, further reduce the sampling number in DNA barcoding.

## Supporting Information

Figure S1 **Comparison of cpDNA (left) and nuclear DNA tree (right) using maximum parsimony (MP) method.** Bootstrap values (>75%) are shown above the branches. Numbers followed taxon names are individual numbers.
(TIF)

Figure S2 **Distribution of inter- and intra-specific K2P distance of combined DNA regions in all studied species.**
(TIF)

Table S1 **List of species used in this study.**
(DOC)

Table S2 **Comparisons of sequence divergence (Kimura 2-parameter distance) among individuals of** ***S. elaeagnifolium*** **and its closest relatives** ***S. hindsianum*** **and** ***S. tridynamum*** **.**
(DOC)
