# Supplementary figures and images for: Species-Specific Identification from Incomplete Sampling: Applying DNA Barcodes to Monitoring Invasive Solanum Plants
Source: PLoS One. 2013 Feb 7;8(2):e55927. doi: 10.1371/journal.pone.0055927 (PMC3567008; doi:10.1371/journal.pone.0055927)

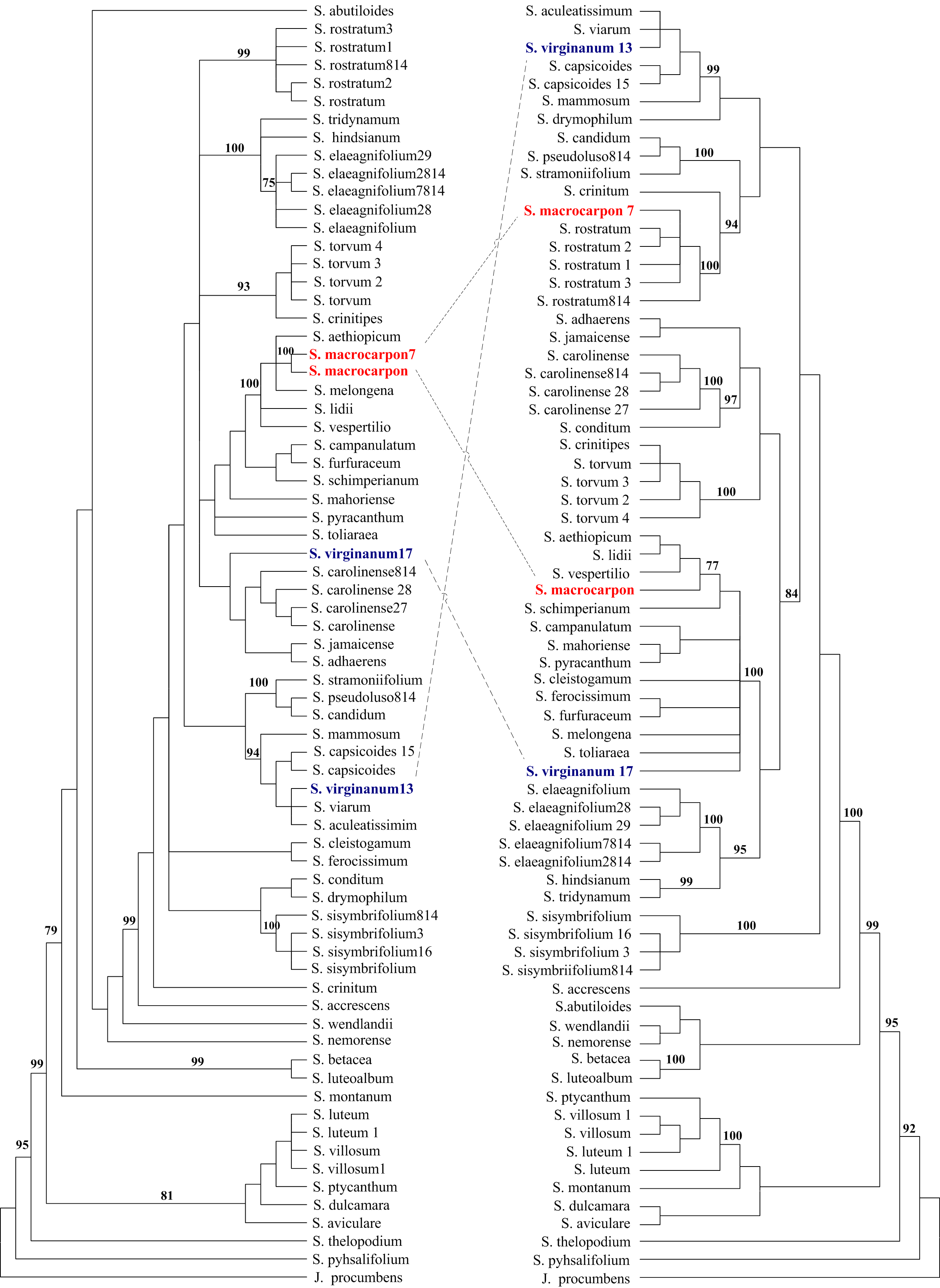

Supplement: Figure S1 — Comparison of cpDNA (left) and nuclear DNA tree (right) using maximum parsimony (MP) method. Bootstrap values (>75%) are shown above the branches. Numbers followed taxon names are individual numbers. (TIF) [file pone.0055927.s001.tif]

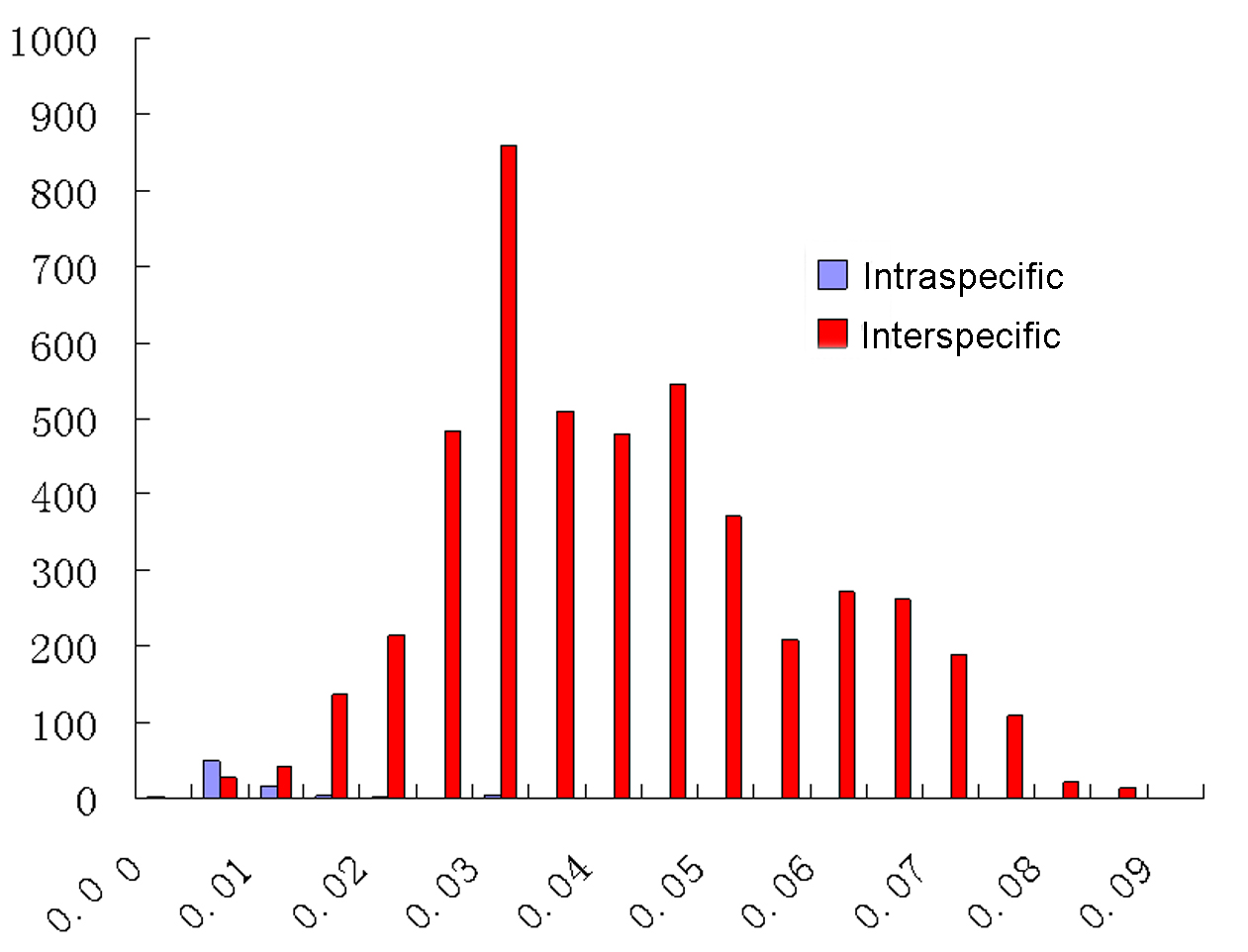

Supplement: Figure S2 — Distribution of inter- and intra-specific K2P distance of combined DNA regions in all studied species. (TIF) [file pone.0055927.s002.tif]
