# Supplementary material for: Species-Specific Identification from Incomplete Sampling: Applying DNA Barcodes to Monitoring Invasive Solanum Plants
Source: PLoS One. 2013 Feb 7;8(2):e55927. doi: 10.1371/journal.pone.0055927 (PMC3567008; doi:10.1371/journal.pone.0055927)
Supplement: Table S1 — List of species used in this study. (DOC) [file pone.0055927.s003.doc]

**Table S1.** List of species used in this study.

| Taxa **^A^** | Provenance | GenBank accession No. | | |
| --- | --- | --- | --- | --- |
|  |  | ndhF | trnS-trnG | Waxy |
| Jaltomata procumbens | GenBank | U47429 | AY998374 | AY996374 |
| Solanum abutiloides | GenBank | U47415.1 | AY555453.1 | [AY562948.1](http://www.ncbi.nlm.nih.gov/nucleotide/45826378?report=genbank&log$=nucltop&blast_rank=1&RID=SWC9UD9J01N) |
| Solanum accrescens | GenBank | AF500795.1 | [AY998375.1](http://www.ncbi.nlm.nih.gov/nucleotide/66865712?report=genbank&log$=nucltop&blast_rank=90&RID=SW75UAEU012) | [AY996375.1](http://www.ncbi.nlm.nih.gov/nucleotide/67037637?report=genbank&log$=nucltop&blast_rank=36&RID=SWC9UD9J01N) |
| Solanum aculeatissimum | Herbiseed | KC469830 | KC469799 | KC469861 |
| Solanum adhaerens | GenBank | AF224061.1 | [AY998376.1](http://www.ncbi.nlm.nih.gov/nucleotide/66865713?report=genbank&log$=nucltop&blast_rank=38&RID=SW79031S013) | [AY996377.1](http://www.ncbi.nlm.nih.gov/nucleotide/67037641?report=genbank&log$=nucltop&blast_rank=1&RID=SWDWKA1V012) |
| Solanum aethiopicum | GenBank | AF500797.11 | [AY998377.1](http://www.ncbi.nlm.nih.gov/nucleotide/66865714?report=genbank&log$=nucltop&blast_rank=10&RID=SW79031S013) | [AY996378.1](http://www.ncbi.nlm.nih.gov/nucleotide/67037643?report=genbank&log$=nucltop&blast_rank=186&RID=SWC9UD9J01N) |
| Solanum aviculare | GenBank | U47418.1 | [AY555458.1](http://www.ncbi.nlm.nih.gov/nucleotide/49065898?report=genbank&log$=nucltop&blast_rank=54&RID=SW75UAEU012) | [AY562952.1](http://www.ncbi.nlm.nih.gov/nucleotide/45826386?report=genbank&log$=nucltop&blast_rank=1&RID=SWEX86G901N) |
| Solanum betacea | GenBank | U47428.1 | [AY998386.1](http://www.ncbi.nlm.nih.gov/nucleotide/66865723?report=genbank&log$=nucltop&blast_rank=1&RID=SW9P0SBR013) | [AY996387.1](http://www.ncbi.nlm.nih.gov/nucleotide/67037661?report=genbank&log$=nucltop&blast_rank=1&RID=SWFCUUSD01S) |
| Solanum campanulatum | GenBank | AF500807.1 | [AY998387.1](http://www.ncbi.nlm.nih.gov/nucleotide/66865724?report=genbank&log$=nucltop&blast_rank=40&RID=SW79031S013) | [AY996388.1](http://www.ncbi.nlm.nih.gov/nucleotide/67037663?report=genbank&log$=nucltop&blast_rank=94&RID=SWDWKA1V012) |
| Solanum candidum | GenBank | AF224072.1 | [AY555459.1](http://www.ncbi.nlm.nih.gov/nucleotide/49065899?report=genbank&log$=nucltop&blast_rank=95&RID=SW79031S013) | [AY562953.1](http://www.ncbi.nlm.nih.gov/nucleotide/45826388?report=genbank&log$=nucltop&blast_rank=1&RID=SWFJT9BK01N) |
| Solanum capsicoides | GenBank | AF500808.1 | [AY555460.1](http://www.ncbi.nlm.nih.gov/nucleotide/49065900?report=genbank&log$=nucltop&blast_rank=76&RID=SW79031S013) | [AY562954.1](http://www.ncbi.nlm.nih.gov/nucleotide/45826390?report=genbank&log$=nucltop&blast_rank=49&RID=SWC9UD9J01N) |
| Solanum capsicoides15 | Herbiseed | KC469831 | KC469800 | KC469862 |
| Solanum carolinense | GenBank | AF500811.1 | [AY998391.1](http://www.ncbi.nlm.nih.gov/nucleotide/66865728?report=genbank&log$=nucltop&blast_rank=1&RID=SWAYUU78016) | [AY996392.1](http://www.ncbi.nlm.nih.gov/nucleotide/67037671?report=genbank&log$=nucltop&blast_rank=119&RID=SWC9UD9J01N) |
| Solanum carolinense27 | Intercepted from soybean imported from American by in Zhangjiagang | KC469832 | KC469801 | KC469863 |
| Solanum carolinense28 | Will country, Illinois, America; Phillippe 38316 | KC469834 | KC469803 | KC469865 |
| Solanum carolinense814 | Intercepted from soybean imported from American by in Zhangjiagang | KC469833 | KC469802 | KC469864 |
| Solanum cleistogamum | GenBank | AF500815.1 | [AY998396.1](http://www.ncbi.nlm.nih.gov/nucleotide/66865733?report=genbank&log$=nucltop&blast_rank=20&RID=SW79031S013) | [AY996397.1](http://www.ncbi.nlm.nih.gov/nucleotide/67037681?report=genbank&log$=nucltop&blast_rank=1&RID=SWVJY9DV01N) |
| Solanum conditum | GenBank | AF500816 | AY998399 | AY996400 |
| Solanum crinitipes | GenBank | AF500817.1 | [AY998401.1](http://www.ncbi.nlm.nih.gov/nucleotide/66865738?report=genbank&log$=nucltop&blast_rank=58&RID=SW79031S013) | [AY996402.1](http://www.ncbi.nlm.nih.gov/nucleotide/67037691?report=genbank&log$=nucltop&blast_rank=54&RID=SWC9UD9J01N) |
| Solanum crinitum | GenBank | AF500818.1 | [AY998402.1](http://www.ncbi.nlm.nih.gov/nucleotide/66865739?report=genbank&log$=nucltop&blast_rank=33&RID=SW79031S013) | [AY996403.1](http://www.ncbi.nlm.nih.gov/nucleotide/67037693?report=genbank&log$=nucltop&blast_rank=73&RID=SWC9UD9J01N) |
| Solanum drymophilum | GenBank | AF500823.1 | [AY998408.1](http://www.ncbi.nlm.nih.gov/nucleotide/66865745?report=genbank&log$=nucltop&blast_rank=52&RID=SW79031S013) | [AY996409.1](http://www.ncbi.nlm.nih.gov/nucleotide/67037705?report=genbank&log$=nucltop&blast_rank=74&RID=SWC9UD9J01N) |
| Solanum dulcamara | Herbiseed | KC469835 | KC469804 | KC469866 |
| Solanum elaeagnifolium | GenBank | AF224067.1 | [AY998411.1](http://www.ncbi.nlm.nih.gov/nucleotide/66865748?report=genbank&log$=nucltop&blast_rank=74&RID=SW79031S013) | [AY996412.1](http://www.ncbi.nlm.nih.gov/nucleotide/67037711?report=genbank&log$=nucltop&blast_rank=1&RID=SWXTW7ZC01N) |
| Solanum elaeagnifolium28 | Intercepted from soybean imported from American by in Zhangjiagang | KC469836 | KC469805 | KC469867 |
| Solanum elaeagnifolium29 | Yavapai country, America; Bartholomew 2430 | KC469837 | KC469806 | KC469868 |
| Solanum elaeagnifolium2814 | Jinan, China; Fan 1201 | KC469839 | KC469808 | KC469870 |
| Solanum elaeagnifolium7814 | B&T world seeds sarl | KC469838 | KC469807 | KC469869 |
| Solanum ferocissimum | GenBank | AF500827 | AY998414 | AY996415 |
| Solanum furfuraceum | GenBank | AF500829.1 | [AY998416.1](http://www.ncbi.nlm.nih.gov/nucleotide/66865753?report=genbank&log$=nucltop&blast_rank=4&RID=SWBKY068012) | [AY996417.1](http://www.ncbi.nlm.nih.gov/nucleotide/67037721?report=genbank&log$=nucltop&blast_rank=136&RID=SWC9UD9J01N) |
| Solanum hindsianum | GenBank | AF500831.1 | [AY998423.1](http://www.ncbi.nlm.nih.gov/nucleotide/66865760?report=genbank&log$=nucltop&blast_rank=32&RID=SW79031S013) | [AY996424.1](http://www.ncbi.nlm.nih.gov/nucleotide/67037735?report=genbank&log$=nucltop&blast_rank=102&RID=SWC9UD9J01N) |
| Solanum jamaicense | GenBank | AF224073.1 | [AY555462.1](http://www.ncbi.nlm.nih.gov/nucleotide/49065902?report=genbank&log$=nucltop&blast_rank=98&RID=SWAYUU78016) | [AY562956.1](http://www.ncbi.nlm.nih.gov/nucleotide/45826394?report=genbank&log$=nucltop&blast_rank=114&RID=SWC9UD9J01N) |
| Solanum lidii | GenBank | AF500839.1 | [AY998433.1](http://www.ncbi.nlm.nih.gov/nucleotide/66865770?report=genbank&log$=nucltop&blast_rank=8&RID=SW79031S013) | [AY996434.1](http://www.ncbi.nlm.nih.gov/nucleotide/67037755?report=genbank&log$=nucltop&blast_rank=177&RID=SWC9UD9J01N) |
| Solanum luteum | Herbiseed | KC469840 | KC469809 | KC469871 |
| Solanum luteum1 | B&T world seeds sarl | KC469841 | KC469810 | KC469872 |
| Solanum luteoalbum | GenBank | U72749.1 | [AY555463.1](http://www.ncbi.nlm.nih.gov/nucleotide/49065903?report=genbank&log$=nucltop&blast_rank=43&RID=SW75UAEU012) | [AY562957.1](http://www.ncbi.nlm.nih.gov/nucleotide/45826396?report=genbank&log$=nucltop&blast_rank=108&RID=SWC9UD9J01N) |
| Solanum macrocarpon | GenBank | AF224068.1 | [AY998435.1](http://www.ncbi.nlm.nih.gov/nucleotide/66865772?report=genbank&log$=nucltop&blast_rank=61&RID=SW79031S013) | [AY996436.1](http://www.ncbi.nlm.nih.gov/nucleotide/67037759?report=genbank&log$=nucltop&blast_rank=48&RID=SWVJY9DV01N) |
| Solanum macrocarpon7 | Fairy lake botanical garden, China;Zhang&Li 1011 | KC469842 | KC469811 | KC469873 |
| Solanum mahoriense | GenBank | AF500841.1 | [AY998436.1](http://www.ncbi.nlm.nih.gov/nucleotide/66865773?report=genbank&log$=nucltop&blast_rank=13&RID=SW79031S013) | [AY996437.1](http://www.ncbi.nlm.nih.gov/nucleotide/67037761?report=genbank&log$=nucltop&blast_rank=1&RID=SWYANPNH01N) |
| Solanum mammosum | GenBank | AF224074.1 | [AY555464.1](http://www.ncbi.nlm.nih.gov/nucleotide/49065904?report=genbank&log$=nucltop&blast_rank=100&RID=SW79031S013) | [AY996438.1](http://www.ncbi.nlm.nih.gov/nucleotide/67037763?report=genbank&log$=nucltop&blast_rank=43&RID=SWC9UD9J01N) |
| Solanum melongena | GenBank | AF224069.1 | [AY555465.1](http://www.ncbi.nlm.nih.gov/nucleotide/49065905?report=genbank&log$=nucltop&blast_rank=4&RID=SW79031S013) | [EU176136.1](http://www.ncbi.nlm.nih.gov/nucleotide/162951642?report=genbank&log$=nucltop&blast_rank=160&RID=SWC9UD9J01N) |
| Solanum montanum | GenBank | AF500844.1 | [AY998443.1](http://www.ncbi.nlm.nih.gov/nucleotide/66865780?report=genbank&log$=nucltop&blast_rank=7&RID=SW75UAEU012) | [AY996443.1](http://www.ncbi.nlm.nih.gov/nucleotide/67037773?report=genbank&log$=nucltop&blast_rank=1&RID=SWYU64C801N) |
| Solanum nemorense | GenBank | AF500847.1 | AY998448.1 | [AY996447.1](http://www.ncbi.nlm.nih.gov/nucleotide/67037781?report=genbank&log$=nucltop&blast_rank=31&RID=SWC9UD9J01N) |
| Solanum pseudolulo814 | B&T world seeds sarl | KC469843 | KC469812 | KC469874 |
| Solanum ptycanthum | Herbiseed | KC469844 | KC469813 | KC469875 |
| Solanum pyhsalifolium | Herbiseed | KC469845 | KC469814 | KC469876 |
| Solanum pyracanthum | GenBank | AF500854.1 | [AY998460.1](http://www.ncbi.nlm.nih.gov/nucleotide/66865797?report=genbank&log$=nucltop&blast_rank=22&RID=SW79031S013) | [AY996459.1](http://www.ncbi.nlm.nih.gov/nucleotide/67037805?report=genbank&log$=nucltop&blast_rank=11&RID=SWYANPNH01N) |
| Solanum rostratum | GenBank | U47424.1 | [AY998465.1](http://www.ncbi.nlm.nih.gov/nucleotide/66865802?report=genbank&log$=nucltop&blast_rank=21&RID=SW79031S013) | [AY562966.1](http://www.ncbi.nlm.nih.gov/nucleotide/45826414?report=genbank&log$=nucltop&blast_rank=35&RID=SWC9UD9J01N) |
| Solanum rostratum1 | BeiJing, China; Fan 0992 | KC469846 | KC469815 | KC469877 |
| Solanum rostratum2 | Intercepted from soybean imported from American by in Zhangjiagang | KC469847 | KC469816 | KC469878 |
| Solanum rostratum3 | Herbiseed | KC469848 | KC469817 | KC469879 |
| Solanum rostratum814 | Shabutai, Neimeng, China; Fan 1092 | KC469849 | KC469818 | KC469880 |
| Solanum schimperianum | GenBank | AF500860.1 | [AY998467.1](http://www.ncbi.nlm.nih.gov/nucleotide/66865804?report=genbank&log$=nucltop&blast_rank=1&RID=SW79031S013) | [AY996465.1](http://www.ncbi.nlm.nih.gov/nucleotide/67037817?report=genbank&log$=nucltop&blast_rank=93&RID=SWYANPNH01N) |
| Solanum sisymbriifolium | GenBank | AF500862.1 | [AY555473.1](http://www.ncbi.nlm.nih.gov/nucleotide/49065913?report=genbank&log$=nucltop&blast_rank=59&RID=SW79031S013) | [AY562967.1](http://www.ncbi.nlm.nih.gov/nucleotide/45826416?report=genbank&log$=nucltop&blast_rank=52&RID=SWDWKA1V012) |
| Solanum sisymbriifolium3 | Mayong, Dongwan, China; Fan 11654 | KC469851 | KC469820 | KC469882 |
| Solanum sisymbriifolium16 | Manoel Ribas Rd, Borboleta Parana; Hatschbach et al., 76775 (herbarium in PE) | KC469850 | KC469819 | KC469881 |
| Solanum sisymbriifolium814 | Quanzhou, China; Zengsizhou 1091 | KC469852 | KC469821 | KC469883 |
| Solanum stramoniifolium | GenBank | AF500863.1 | [AY555476.1](http://www.ncbi.nlm.nih.gov/nucleotide/49065916?report=genbank&log$=nucltop&blast_rank=79&RID=SW79031S013) | [AY562970.1](http://www.ncbi.nlm.nih.gov/nucleotide/45826422?report=genbank&log$=nucltop&blast_rank=1&RID=SWYZAB0K01N) |
| Solanum thelopodium | GenBank | AF500865.1 | AY998472.1 | [AY996471.1](http://www.ncbi.nlm.nih.gov/nucleotide/67037829?report=genbank&log$=nucltop&blast_rank=1&RID=SWZ413E201N) |
| Solanum toliaraea | GenBank | AF500866.1 | [AY998473.1](http://www.ncbi.nlm.nih.gov/nucleotide/66865810?report=genbank&log$=nucltop&blast_rank=16&RID=SW79031S013) | AY996472.1 |
| Solanum torvum | GenBank | L76286.1 | [AY555478.1](http://www.ncbi.nlm.nih.gov/nucleotide/49065918?report=genbank&log$=nucltop&blast_rank=62&RID=SW79031S013) | [AY562972.1](http://www.ncbi.nlm.nih.gov/nucleotide/45826426?report=genbank&log$=nucltop&blast_rank=48&RID=SWC9UD9J01N) |
| Solanum torvum2 | Mayong, Dongwan, China; Fan 11681 | KC469853 | KC469822 | KC469884 |
| Solanum torvum3 | Okinawa,Japan;unknown  1992-6-20(herbarium in PE) | KC469854 | KC469823 | KC469885 |
| Solanum torvum4 | B&T world seeds sarl | KC469855 | KC469824 | KC469886 |
| Solanum tridynamum | GenBank | AF500867.1 | [AY998475.1](http://www.ncbi.nlm.nih.gov/nucleotide/66865812?report=genbank&log$=nucltop&blast_rank=80&RID=SW79031S013) | AY996474.1 |
| Solanum vespertilio | GenBank | AF224070.1 | [AY998477.1](http://www.ncbi.nlm.nih.gov/nucleotide/66865814?report=genbank&log$=nucltop&blast_rank=20&RID=SWBKY068012) | [AY996476.1](http://www.ncbi.nlm.nih.gov/nucleotide/67037839?report=genbank&log$=nucltop&blast_rank=36&RID=SWVJY9DV01N) |
| Solanum viarum | Herbiseed | KC469856 | KC469825 | KC469887 |
| Solanum villosum | Herbiseed | KC469857 | KC469826 | KC469888 |
| Solanum villosum1 | B&T world seeds sarl | KC469858 | KC469827 | KC469889 |
| Solanum virginanum13 | Guangzhou, China; Zhang&Fan1024 | KC469859 | KC469828 | KC469890 |
| Solanum virginanum17 | B&T world seeds sarl | KC469860 | KC469829 | KC469891 |
| Solanum wendlandii | GenBank | U47427.1 | [AY555481.1](http://www.ncbi.nlm.nih.gov/nucleotide/49065921?report=genbank&log$=nucltop&blast_rank=17&RID=SW75UAEU012) | AY562974.1 |

^A^ Numbers followed taxon names are individual numbers.
