# Supplementary material for: Species-Specific Identification from Incomplete Sampling: Applying DNA Barcodes to Monitoring Invasive Solanum Plants
Source: PLoS One. 2013 Feb 7;8(2):e55927. doi: 10.1371/journal.pone.0055927 (PMC3567008; doi:10.1371/journal.pone.0055927)
Supplement: Table S2 — Comparisons of sequence divergence (Kimura 2-parameter distance) among individuals of S. elaeagnifolium and its closest relatives S. hindsianum and S. tridynamum . (DOC) [file pone.0055927.s004.doc]

**Table S2**. Comparisons of sequence divergence (Kimura 2-parameter distance) among individuals of *S. elaeagnifolium* and its closest relatives *S. hindsianum* and *S. tridynamum*.

| Species pair | | Sequences divergence |
| --- | --- | --- |
| Solanum hindsianum | Solanum tridynamum | 0.683% |
| Solanum tridynamum | Solanum hindsianum | 0.683% |
| Solanum elaeagnifolium | Solanum tridynamum | 1.3155% |
| Solanum tridynamum | Solanum elaeagnifolium | 1.3155% |
| Solanum elaeagnifolium | Solanum tridynamum | 1.3408% |
| Solanum elaeagnifolium | Solanum hindsianum | 1.3408% |
| Solanum hindsianum | Solanum elaeagnifolium | 1.3408% |
| Solanum tridynamum | Solanum elaeagnifolium | 1.3408% |
| Solanum elaeagnifolium | Solanum hindsianum | 1.3661% |
| Solanum hindsianum | Solanum elaeagnifolium | 1.3661% |
| Solanum elaeagnifolium | Solanum tridynamum | 1.3913% |
| Solanum tridynamum | Solanum elaeagnifolium | 1.3913% |
| Solanum elaeagnifolium | Solanum hindsianum | 1.4166% |
| Solanum elaeagnifolium | Solanum tridynamum | 1.4166% |
| Solanum hindsianum | Solanum elaeagnifolium | 1.4166% |
| Solanum tridynamum | Solanum elaeagnifolium | 1.4166% |
| Solanum elaeagnifolium | Solanum hindsianum | 1.4419% |
| Solanum hindsianum | Solanum elaeagnifolium | 1.4419% |
| Solanum elaeagnifolium | Solanum tridynamum | 1.4672% |
| Solanum tridynamum | Solanum elaeagnifolium | 1.4672% |
| Solanum elaeagnifolium | Solanum hindsianum | 1.4925% |
| Solanum hindsianum | Solanum elaeagnifolium | 1.4925% |
| Solanum elaeagnifolium | Solanum elaeagnifolium | 0.7589% |
| Solanum elaeagnifolium | Solanum elaeagnifolium | 0.7589% |
| Solanum elaeagnifolium | Solanum elaeagnifolium | 0.7336% |
| Solanum elaeagnifolium | Solanum elaeagnifolium | 0.7336% |
| Solanum elaeagnifolium | Solanum elaeagnifolium | 0.6324% |
| Solanum elaeagnifolium | Solanum elaeagnifolium | 0.6324% |
| Solanum elaeagnifolium | Solanum elaeagnifolium | 0.5565% |
| Solanum elaeagnifolium | Solanum elaeagnifolium | 0.5565% |
| Solanum elaeagnifolium | Solanum elaeagnifolium | 0.5312% |
| Solanum elaeagnifolium | Solanum elaeagnifolium | 0.5312% |
| Solanum elaeagnifolium | Solanum elaeagnifolium | 0.4806% |
| Solanum elaeagnifolium | Solanum elaeagnifolium | 0.4806% |
| Solanum elaeagnifolium | Solanum elaeagnifolium | 0.4048% |
| Solanum elaeagnifolium | Solanum elaeagnifolium | 0.4048% |
| Solanum elaeagnifolium | Solanum elaeagnifolium | 0.4301% |
| Solanum elaeagnifolium | Solanum elaeagnifolium | 0.4301% |
| Solanum elaeagnifolium | Solanum elaeagnifolium | 0.0253% |
| Solanum elaeagnifolium | Solanum elaeagnifolium | 0.0253% |
| Solanum elaeagnifolium | Solanum elaeagnifolium | 0.1518% |
| Solanum elaeagnifolium | Solanum elaeagnifolium | 0.1518% |
